# The efficacy and safety of corticosteroids in treating severe COVID-19 patients: A meta-analysis with trial sequential analysis

**DOI:** 10.12669/pjms.41.5.9545

**Published:** 2025-05

**Authors:** Yang Zhang, Jiepin Hu, Qiong Xu, Feng Zhang, Chunlei Sun, Kailei Liu

**Affiliations:** 1 Dr. Yang Zhang, Department of Pharmacy, Zhejiang Putuo Hospital, Zhejiang, China. Branch of Sir Run Run Shaw Hospital, Zhejiang Unversity School of Medicine, Zhejiang, China; 2 Dr. Jiepin Hu, Department of Pharmacy, Zhejiang Putuo Hospital, Zhejiang, China. Branch of Sir Run Run Shaw Hospital, Zhejiang Unversity School of Medicine, Zhejiang, China; 3 Dr. Qiong Xu, Department of Pharmacy, Zhejiang Putuo Hospital, Zhejiang, China. Branch of Sir Run Run Shaw Hospital, Zhejiang Unversity School of Medicine, Zhejiang, China; 4 Prof. Feng Zhang, Department of Pharmacy, Zhejiang Putuo Hospital, Zhejiang, China. Branch of Sir Run Run Shaw Hospital, Zhejiang Unversity School of Medicine, Zhejiang, China; 5 Prof. Chunlei Sun, Department of Intensive Care Unit, Zhejiang Putuo Hospital, Zhejiang, China; 6 Prof. Kailei Liu, Department of Pharmacy, Zhejiang Putuo Hospital, Zhejiang, China. Branch of Sir Run Run Shaw Hospital, Zhejiang Unversity School of Medicine, Zhejiang, China

**Keywords:** Corticosteroids, Covid-19, Glucocorticoids, Novel coronavirus pneumonia, SARS-CoV-2

## Abstract

**Background and Objective:**

Corticosteroids (CSs) are often administered for coronavirus disease 2019 (COVID-19); however, the advantages and disadvantages of CSs remain controversial. Accordingly, we performed a meta-analysis with trial sequential analysis (TSA) to ascertain the efficacy and safety of CSs in treating COVID-19.

**Methods:**

From their inception until April 2023, PubMed, Embase, The Cochrane Library, Web of Science, and China Biology Medicine(CBM) were searched to gather randomized controlled trials on the administration of CSs for COVID-19 treatment. Following the literature screening according to the inclusion criteria, data extraction and quality assessment were conducted by two reviewers, and then we conducted a meta-analysis with trial sequential analysis using RevMan 5.4 and TSA v0.9, respectively. This meta-analysis adhered to the Preferred Reporting Items for Systematic Reviews and Meta-analyses (PRISMA) guidelines, and the study protocol was registered with PROSPERO (CRD42023458633).

**Results:**

A total of 6,077 literatures were obtained through the search, and 14 studies were finally included for quantitative meta-analysis. The meta-analysis revealed that the all-cause mortality in the group treated with CSs and standard treatment was significantly lower than that in the control group that received only standard treatment (RR 0.89, 95% CI: 0.82–0.96, P=0.002), with a statistically significant difference. However, the TSA analysis revealed that the cumulative Z value exceeded the traditional boundary value yet did not surpass the TSA boundary value, indicating a possibility of false positive outcomes in the meta-analysis. The incidence of adverse reactions in the group receiving CSs treatment was higher than that in the control group, but the distinction was not statistically significant (RR 1.02, 95% CI: 0.64–1.63, P=0.93).

**Conclusion:**

CSs appear to be effective and safe in decreasing the overall mortality of patients who suffer from severe COVID-19; however, further assessment is required to determine adverse reactions and improvements in clinical symptoms associated with CS administration.

## INTRODUCTION

COVID-19, or Novel Coronavirus Pneumonia, is an infectious respiratory disease caused by SARS-CoV-2 (Severely Acute Respiratory Syndrome Coronavirus 2).^1^ Infection with this enveloped RNA virus can cause damage to multiple systems, including the respiratory, digestive, and nervous systems. Typical symptoms include fever, cough, malaise, dyspnea, headache, arthralgia, and diarrhea. Some patients with severe and critical illnesses may experience multi-organ failure, which can be life-threatening.^2^,^3^ Since its initial outbreak four years ago, it has become a significant public health issue worldwide.^4^ Despite international efforts to control the spread, the number of cases and deaths caused by the novel coronavirus has increased globally.^5^,^6^ The emergence of the highly contagious SARS-CoV-2 variant, such as the Delta and Omicron, further compounded the epidemic prevention and control challenges.^7^ Research has demonstrated that individuals aged 65 or above, as well as those with specific underlying conditions, including cancer, chronic obstructive pulmonary disease, chronic kidney disease, heart disease, obesity, sickle cell disease or diabetes, are at an elevated risk of developing severe illness, being hospitalized and even experiencing mortality.^8^-^10^

The systemic inflammatory response in patients who suffered from COVID-19, caused by the excessive release of cytokines and inflammatory mediators, may result in lung injury and, potentially, progression to acute respiratory distress syndrome. Corticosteroids (CSs) possess strong anti-inflammatory properties and may mitigate or prevent these harmful responses by regulating cytokine release.^11^

Healthcare professionals have refocused their efforts on the administration of CSs in severe COVID-19 cases because of the widespread impact of the COVID-19 pandemic, the limited availability of effective treatments, the recognized anti-inflammatory properties of CSs, and preliminary investigations into the administration of CSs in SARS-CoV and MERS-CoV infections.^12^ Nevertheless, the advantages and disadvantages of CS therapy lack conclusive evidence-based recommendations, leading to ongoing debates regarding its application in clinical settings.^13^,^14^

Therefore, we undertook a systematic review using meta-analysis and trial sequential analysis (TSA) to augment comprehension regarding the efficacy and safety of CSs in treating patients afflicted with COVID-19, specifically those experiencing severe manifestations.^15^ Our principal objective was to furnish a substantiated rationale for formulating and modifying clinical practices and guidelines based on empirical evidence.

## METHODS

Our study was performed in accordance with the guidelines outlined in the Preferred Reporting Items for Systematic Reviews and Meta-Analysis (PRISMA) guidelines, and the study protocol was registered with PROSPERO (CRD42023458633). We defined the severity of COVID-19 according to the World Health Organization (WHO) issued Guidelines for the Clinical Management of COVID-19.

### Inclusion Criteria:

The inclusion criteria for this study consisted of the following:

- A randomized controlled trial (RCT).
- Individuals aged 18 years and older, regardless of gender or race, who satisfied the diagnostic criteria for COVID-19 and met the WHO disease severity and progression scores of ≥4 for COVID-19 patients.^16^
- CSs (administered orally, intravenously, or via inhalation) in conjunction with standard treatment versus standard treatment alone. The data to be considered should encompass demographic information and quantitative measures, such as all-cause mortality and incidence of adverse reactions.

### Exclusion criteria:

The exclusion criteria for this study encompassed the following:

- Studies involving the combination of CSs with other anti-inflammatory agents (e.g., anabolic agents) were excluded.
- Investigations assessing the efficacy of CSs in the treatment of other coronaviral diseases, such as SARS or Middle East Respiratory Syndrome.
- Replicated published studies or studies lacking relevant outcome reporting.
- Studies involving patients with mild or asymptomatic conditions.
- Case reports, editorials, and non-comparative observational studies were not considered.

### Search strategy:

We conducted a comprehensive search across multiple databases, including PubMed, Web of Science, Embase, The Cochrane Library, and CBM, to identify randomized controlled trials examining the efficacy of CS treatment for severe cases of COVID-19. The search period spanned from the inception of each database up until April 2023. The search strategy used a combination of subject terms and free words. The search terms used in both Chinese and English included neocoronavirus, neocoronavirus pneumonia, COVID-19, the novel coronavirus disease of 2019, glucocorticosteroids, corticosteroids, hydrocortisone, prednisone, methylprednisolone, and dexamethasone.

### Data extraction:

Two researchers conducted a comprehensive literature review, employing established criteria to extract and cross-verify data, and any discrepancies were resolved through discussion or with the assistance of a third party. The screening process involved an initial evaluation of the paper’s title to eliminate irrelevant material, followed by a thorough examination of the abstract and full text to determine the final selection for further analysis.

The data extraction procedure encompassed the following steps: first, furnishing imperative particulars of the studies, including the study title, primary author’s name, publication date, and study location. Additionally, the procedure incorporates baseline characteristics of the study population, encompassing sample size, gender distribution, and age demographics. Furthermore, it is essential to provide comprehensive information regarding the intervention, containing the specific CSs used, the dosage administered, and the mode of administration implemented for each patient group. Lastly, presenting the outcome indicators and measurement data of interest is imperative to ensure accurate reporting.

### Quality assessment:

Two investigators independently evaluated the risk of bias for the included studies, and the findings were verified through cross-checking. We used the recommended RCT risk of bias evaluation tool from the Cochrane Handbook to assess the risk of bias.^17^ The evaluation encompassed an assessment of the study’s quality, which was conducted by considering the following criteria: (i) the randomization method, (ii) the concealment of allocation, (iii) the implementation of blinding for both patients and investigators, (iv) the implementation of blinding for outcome assessors, (v) the completeness of outcome data, (vi) the selective reporting of study results, and (vii) the identification of any additional sources of bias.

### Statistical analysis:

The statistical analysis was conducted with RevMan 5.4 software. Mean difference (MD) was used as the effect analysis statistic for measures, whereas risk ratio (RR) was used for dichotomous variables. 95% confidence intervals (CI) were provided for each effect. The heterogeneity among the outcomes of the included studies was examined using the chi-square test (with a significance level of α=0.1), and the degree of heterogeneity was assessed by combining I². If the results of the included studies did not exhibit statistical heterogeneity, a fixed-effects model was used for meta-analysis. After excluding the effect of significant clinical heterogeneity, a random-effects model was used for meta-analysis. The significance level for meta-analysis was established at α=0.05. To account for substantial clinical heterogeneity, we used subgroup analysis.

Furthermore, funnel plots were generated to evaluate publication bias. Abbreviations of technical terms were clarified upon initial usage. Lastly, TSA was conducted using TSA v0.9 software, which was developed by the Copenhagen Clinical Trials Centre in Denmark.^18^ TSA—a variant of cumulative meta-analysis—offers a means to tackle the problem of random error (false negative/false positive outcomes) encountered in conventional meta-analysis when undergoing repeated updates. Additionally, it enables the determination of the necessary sample size for attaining a precise conclusion.

## RESULTS

### Literature search results:

**Fig.1:**
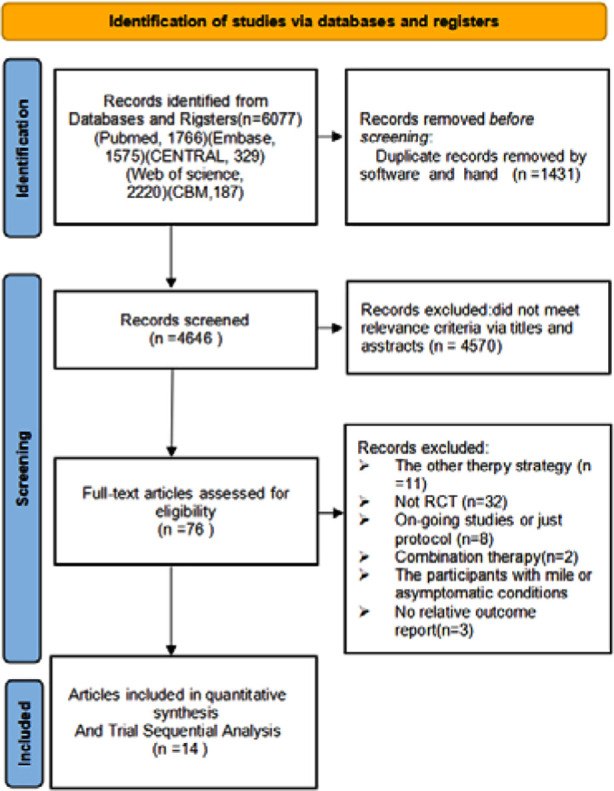
PRISMA flowchart detailing literature search and screening strategy.

The search strategy implemented resulted in the initial retrieval of 6,077 documents. After eliminating duplicates and a preliminary assessment of titles and abstracts, 4,646 documents remained. Subsequent examination of the full texts resulted in 76 documents being selected for re-screening. Ultimately, 14 studies were subjected to quantitative meta-analysis, as indicated by the reference range.^19^-^32^ The process of literature screening and its outcomes are visually represented in Fig.1.

### Basic Characteristics of Included Studies and Risk of Bias Results:

The fundamental traits of the included studies are presented in Table-I.

**Table-I:** Basic characteristics of included studies.

Study ID	Location	Age(mean±standard or Median (interquartile range)		Gender(male/female)		Sample size		Intervention details		Outcome measured	Score
		T	C	T	C	T	C	T	C		
Angus 2020	Australia, Canada, France, Ireland, New Zealand, the Netherlands, UK, USA	Ta:60.4±11.6 Tb:59.5±12.7	59.9 ±14.6	Ta:98/ 39 Tb:103/43	72 /29	295	108	hydrocortisone, IV, 150 mg/d, Ta: 7 d; Tb: 28 d (if in shock)	Standard care	①②	≥6
Dequin 2020	France	63.1 (51.5-70.8)	66.3 (53.5-72.7)	54/ 22	50/23	76	73	Hydrocortisone, IV, 200 mg/d: 7 d; 100 mg/d: 4 d; 50 mg/d: 3 d	Standard care	①②	≥5
Edalatifard 2020	Iran	55.8±16.35	61.7±16.62	24 /10	15/ 13	34	28	Methylprednisolone, IV, 250 mg/d:3 d	Standard care	①②	5-6
Jeronimo 2020	Brazil	54±15	57±15	68 /126	71/128	194	199	Methylprednisolone (as sodium succinate), IV, 1 mg/kg bw: 5 d	Standard care	①	5-9
Tang 2021	China	57 (49–67)	55 (38–65)	21/22	20 /23	43	43	Methylprednisolone, IV, 1 mg/kg bw: 7 d	Standard care	①②	4-6
Corral-Gudino 2021	Spain	73±11	66±12	23/12	16/13	35	29	Methylprednisolone, IV, 80 mg/d : 3 d, 40 mg/d: 3 d	Standard care	①②	5-6
Tomazini 2020	Brazil	60.1±15.8	62.7 ±13.1	90 /61	97 /51	151	148	Dexamethasone, IV, 20 mg/d: 5 d; 10 mg/d: 5 d	Standard care	①②	7-9
Horby 2021	UK	66.9 ± 15.4	65.8± 15.8	1338/766	2749/1572	2104	4321	Dexamethasone, IV or oral, 6 mg/d : 10 d	Standard care	①	4-9
Jamaati 2021	Iran	Ts:54(37–63) Tn:63(55.5–72.5)	Cs:61.5(54–62) Cn:67 (48–73)	Ts:6 /3 Tn:12 /4	Cs:7/3 Cn:11/4	25	25	Dexamethasone, IV,20 mg/d: 5 d; 10 mg/d: 5 d	Standard care	①	≥5
Munch 2021	Denmark	59 (52-74)	62 (55-71)	14/2	10/4	16	14	Hydrocortisone,IV,200 mg/d: 7 d or until discharge	Standard care	①②	≥5
Ghanei 2022	Iran	58.2±17.2	57.6±15.6	57/59	55 /55	116	110	Prednisone, oral or IV, 25 mg/d, 5 d	Azithromycin, oral,250mg/d,5 d	①②	≥5
Emma 2022	Netherlands	51(42.5-59.3)	46(39.5-55.0)	18/39	24/34	58	57	Prednisone, oral, 40 mg/d, 10 d	Standard care	②	≥5
Agusti 2022	Spain	50.6±13.7	51.6±13.8	24/34	32/30	58	62	Budesonide, inhale, 400 μg once, bid, 15 d	Standard care	①②	≥4
Yu 2022	UK	64.7±7.3	64.5±7.7	404/429	431/455	789	1069	Budesonide, inhale, 800 μg once, bid, 14 d	Standard care	①②	≥4

Note: (1) T: test group, C: control group, where Ta represents the fixed-dose intervention group, Tb represents the shock-dependent intervention group, Ts represents the test group survivor group, Tn represents the test group death group, Cs represents the control group survivor group, and Cn represents the control group death group. (2) IV denotes intravenous injection (route of administration), bid signifies administration twice daily, and bw indicates body weight. Outcome indicators encompass all-cause mortality, the occurrence of adverse reactions. The score pertains to the scoring scale devised by the World Health Organization, which evaluates the severity and advancement of COVID-19 in afflicted individuals.

### Meta-analysis Results:

### All-Cause Mortality:

**Fig.2:**
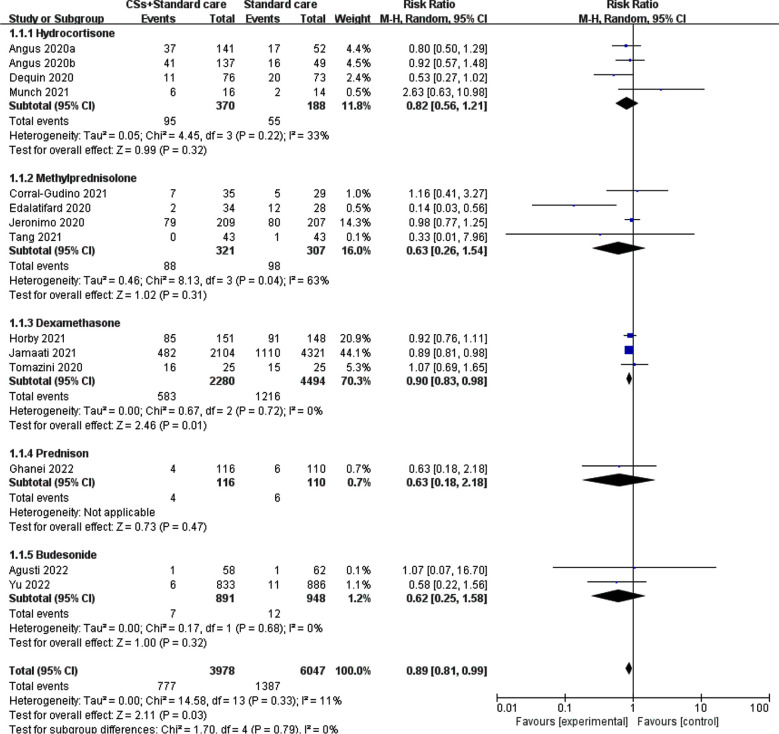
Meta-analysis of CSs combined with standard treatment vs. standard treatment for all-cause mortality.

All-cause mortality was observed in 13 RCTs included in this study which explored the benefits and harms of dexamethasone and methylprednisolone.^19^-^31^ The heterogeneity test conducted in the present analysis revealed a decrease in variation among the studies (I²=7%, P=0.38). The meta-analysis using the fixed-effects model manifested a significant reduction in all-cause mortality among patients who received CS treatment in conjunction with standard therapy, as opposed to those who solely received standard treatment (RR=0.89, 95% CI: 0.82–0.96, P=0.002) (Fig.2). Subgroup analyses based on the specific CSs administered in each study revealed notable heterogeneity, with hydrocortisone and methylprednisolone exhibiting I² values of 33% and 63%, respectively. The meta-analysis findings indicated that patients who received a combination of hydrocortisone, methylprednisolone, prednisone, and budesonide had lower all-cause mortality rates than those who received the standard treatment alone, although this disparity did not get to statistical significance. This phenomenon may be ascribed to insufficient sample size, disparities in the severity of patients’ neocoronitis diagnosis, the dosage and duration of administration, and the timing of outcome indicator measurements. Notably, the meta-analysis revealed that the dexamethasone group exhibited a statistically significant reduction in all-cause mortality (RR=0.90, 95% CI: 0.83–0.98, P=0.01).

### Incidence of adverse reactions:

**Fig.3:**
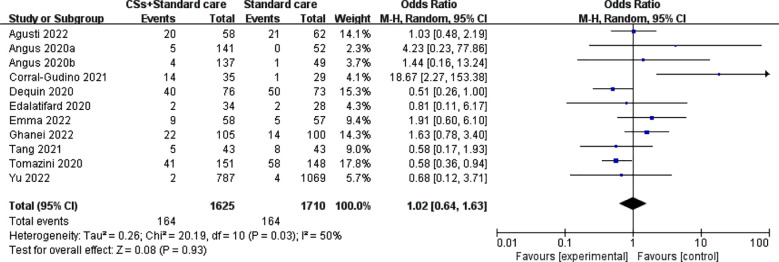
Meta-analysis of CSs combined with standard treatment vs. standard treatment for incidence of ADR.

Ten studies^19^-^21^, ^23^-^25^, ^29^-^32^ have assessed the incidence of adverse reactions in patients undergoing CSs treatment. The analysis revealed substantial heterogeneity among the included studies (I²=50%, P=0.03). The random-effects model meta-analysis indicated that the group receiving CSs treatment had a lower incidence of adverse reactions than the control group. However, this difference did not reach statistical significance (RR=1.02, 95% CI: 0.64–1.63, P=0.93) (Fig.3).

### Analysis of Publication Bias and Risk of Bias of the included studies:

We used funnel plots to assess the presence of publication bias in the comparison of CSs combined with standard treatment versus standard treatment. The analysis of publication bias showed that the distribution of study sites in the funnel plot was not entirely symmetrical, illustrating the presence of potential publication bias. The outcomes of the methodological quality evaluation of the included studies which displays the risk of bias and the result of publication bias are shown in Supplementary Materials.

**Supplementary Fig.1:**
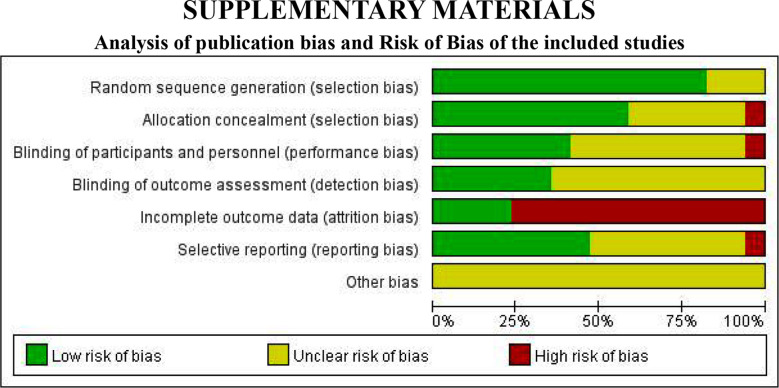
Risk of bias map for included studies.

**Supplementary Fig.2:**
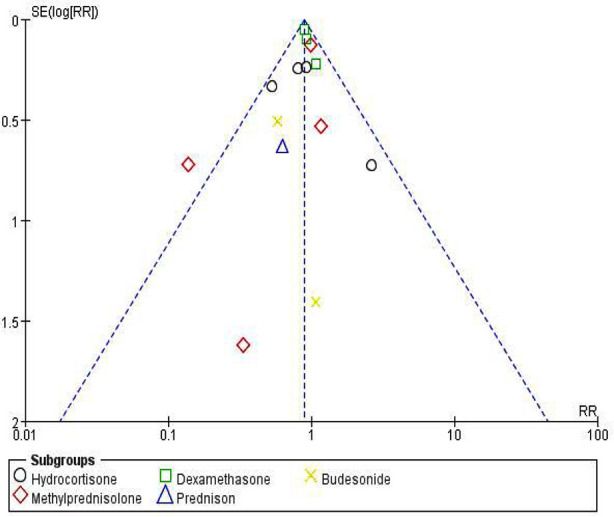
TSA results for CSs combined with standard treatment compared with standard treatment for all-cause mortality.

### TSA Analysis Results:

**Fig.4:**
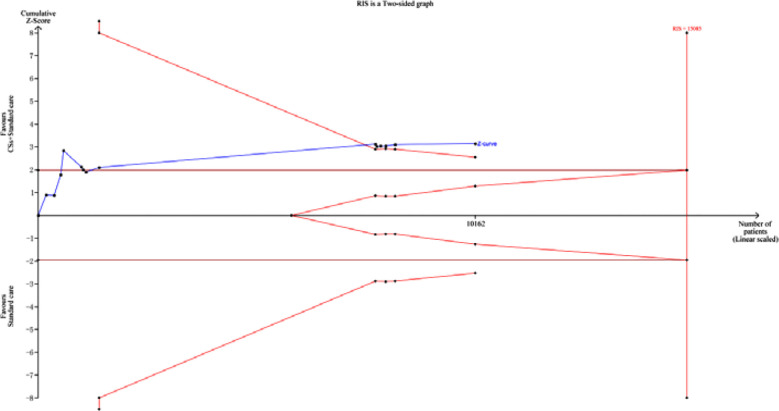
Funnel plot of all-cause mortality for CSs combined with standard treatment compared with standard treatment.

The cumulative Z value surpassed the conventional thresholds upon inclusion of the ninth study in Fig.4. Nevertheless, it did not surpass the TSA thresholds or attain the RIS, thereby not eliminating the possibility of false-positive results in the Meta-analysis. This finding necessitates further clinical trials with substantial sample sizes and rigorous quality criteria to validate the dependability of the results.

## DISCUSSION

The Coronavirus infection is characterized by not only pulmonary impairment but also a persistent elevation in cytokine and chemokine levels, commonly known as an “inflammatory storm.” In severe instances, this immune response can result in multi-organ failure, compromised immune function, and potentially fatal outcomes.^33^-^34^ Approximately 5% of critically ill patients are documented to experience morbidity, whereas a concerning 49% succumb to mortality.^35^ And it is worth to be mentioned is that the total leucocyte count, absolute neutrophil count and neutrophil lymphocyte ratio could be used to predict mortality and morbidity in severe COVID-19 patients.^36^ Furthermore, the medical condition of patients classified as severe and critical demonstrates a rapid decline, resulting in an unfavorable prognosis.^37^,^38^ Regrettably, targeted pharmacological interventions are absent.^39^,^40^ A variety of administrative measures introduced worldwide to treat the COVID-19 at the outset of pandemic without any evidence of therapeutic benefit which appealed to create and deliver the evidence-based recommendations.^41^ Notably, patients experience a swift deterioration in their condition, leading to a bleak prognosis without any specific pharmaceutical remedies accessible. CSs are commonly used to treat various inflammatory lung diseases; however, their administration may worsen existing infections, prolong the duration of the condition, and elicit other adverse reactions. Prolonged administration of high doses of CSs can lead to fractures, necrosis of the femoral head, hyperglycemia, gastrointestinal bleeding, and other severe complications. Consequently, the administration of CSs in clinical settings has consistently been regarded as a double-edged sword.^42^ The guidelines published by the World Health Organization (WHO) and China propose that systemic CS therapy in low doses and for a limited duration could be considered for patients with COVID-19.^43^ However, the current body of evidence-based medicine is inadequate to support this recommendation conclusively. Consequently, the administration of CSs in patients with COVID-19 remains debatable. The present study systematically assessed and conducted a meta-analysis on the effectiveness and safety of CS treatment for neocoronary pneumonia.

**Table:** 

2. Search strategy (take Pubmed as an example)
*Condition search*
#1 exp COVID-19/
#2 Neocoronavirus pneumonia.mp.
#3 The novel coronavirus disease of 2019.mp.
#4 Exp Corticosteroids/
#5 Glucocorticosteroids.mp.
#6 Hydrocortisone.mp.
#7 Prednisone.mp.
#8 Methylprednisolone.mp.
#9 Dexamethasone.mp.
#10 #1 or #2 or #3
#11 #4 or #5 or #6 or #7 or #8 or #9
#12 #10 and #11
*Filter to identify RCTs*
#13 exp “clinical trial [publication type]”/
#14 (randomised or randomised).ab,ti.
#15 #13 or #14
#16 #12 and #15

Out of the 14 studies examined in this analysis, 12 studies have reported a positive enhancement in clinical signs. Various areas showed improvements in clinical signs, including the duration of ventilatory support independence, clinical recovery time, hospital stay duration, enhancement in the Glasgow Coma Score (GCS), and the occurrence of emergency care associated with COVID-19. Angus et al. revealed a potential advantage in the administration of hydrocortisone to extend the duration of ventilator independence in patients with severe COVID-19 by day 21. However, the premature termination of the trial hindered the establishment of definitive conclusions.^19^ By contrast, Deguin et al. reported that the administration of low-dose hydrocortisone in critically ill COVID-19 patients with acute respiratory failure did not result in a significant reduction in the occurrence of treatment failure, defined as mortality, by day 21. The criteria for day treatment failure encompass mortality or the need for ongoing respiratory support. The trial was prematurely halted and did not yield conclusive findings.^20^ Edalatifard et al. provided evidence that administering methylprednisolone pulses with GCS to COVID-19 patients experiencing severe respiratory failure significantly enhanced clinical indicators.^21^ Conversely, Jammati et al. observed that 92% of individuals in the dexamethasone group and 96% in the control group necessitated non-invasive ventilation, with no statistically significant distinction (p=0.500).^27^ Munch et al. demonstrated no statistically significant disparity between the hydrocortisone and placebo cohorts concerning the median count of ventilator-support-free days on day 28 (7 vs. 10, 95% CI: -9.5 to 7.3, P=0.79).^28^ Conversely, Ramakrishnan et al. ascertained that the early administration of budesonide through inhalation diminished the probability of necessitating urgent medical attention and expedited the duration until clinical recuperation in individuals with early manifestations of COVID-19.^44^ Tomazini et al. demonstrated that the administration of dexamethasone in conjunction with standard therapy resulted in a significant increase in the number of days free from ventilator support and survival days at 28 days for patients with COVID-19 and moderate or severe ARDS, compared to the administration of dexamethasone alone.^25^ Similarly, Ranjbar et al. found that the group receiving methylprednisolone exhibited significant clinical improvement over the dexamethasone control group at day five (4.02 vs. 5.21, P=0.002) and day 10 (2.90 vs. 4.71, P=0.001) after admission. The group treated with methylprednisolone had an average hospital stay of 7.43±3.64 days, whereas the group treated with dexamethasone had an average hospital stay of 10.52±5.47 days (P=0.015).

The methylprednisolone trial group had significantly lower ventilator requirements (18.2%) than the dexamethasone control group (38.1%, P=0.040).^45^ Ghanei et al. demonstrated that the prednisone trial group had a significantly shorter hospital stay than the lopinavir/ritonavir treatment group (P=0.028).^31^ Agusti et al. have provided evidence that administering inhaled budesonide resulted in a deceleration of disease progression.^30^ Similarly, Yu et al. found that individuals in the budesonide group experienced a significantly shorter time to first self-reported recovery compared with those in the usual care group (2.94 vs. 11.8, HR=1.21, 95% CI: 1.08–1.36). Furthermore, the prednisone group exhibited a significantly shorter mean hospital stay duration than the control groups, and both treatment groups demonstrated similar improvements in olfactory function after 12 weeks of treatment initiation.^29^

The findings indicate that CSs have the potential to decrease overall mortality and extend survival among individuals diagnosed with severe neocoronary pneumonia. Nevertheless, the TSA highlights the potential for erroneous positive outcomes within the meta-analysis. Consequently, we advocate for additional investigation in this domain, especially the relationship and association between the administration of CSs and long-term COVID-19.^46^

### Limitations:

This study possesses several limitations. First, including a relatively small number of studies and a small sample size is noteworthy. Furthermore, the study has not yet attained the requisite effective sample size of 12,080 prescribed by RIS standards. The CSs used in these studies exhibited variations in both type and dosage. Although the types were subjected to subgroup analysis, the dose and duration of treatment courses were not, impeding the execution of a dose-response meta-analysis. The selected studies in the analysis exhibited variations in the chosen outcome indicators, and the assessment period for all-cause mortality differed, ranging from 21 to 90 days, among other durations. Additionally, some included studies did not adhere strictly to the original trial protocols when reporting outcome indicators and data, which introduces the possibility of selective bias in the results. Moreover, the systematic evaluation did not encompass a search for relevant grey literature, potentially leading to publication bias.

## CONCLUSION

In summary, administering CSs may potentially reduce overall mortality rates, although additional assessment is warranted to ascertain the prevalence of adverse effects and the enhancement of clinical manifestations. Due to the limited quantity and quality of the included studies, these findings may need to be validated by supplementary high-quality, extensive clinical trials.

### Recommendation:

Corticosteroids is recommended in treating severe COVID-19 patients especially the one who need oxygen or mechanical ventilation while strict monitoring of their adverse effects is required.

### Authors Contribution:

**YZ** conceived, designed and did statistical analysis & editing of manuscript, is responsible for integrity of research. **JPH ,QX, FZ& CS did** data collection and manuscript writing. **KLL** Critical review and final approval of manuscript. All authors have approved the final version to be published.
